# Antibiotic Resistance and Biofilm Infections in the NICUs and Methods to Combat It

**DOI:** 10.3390/antibiotics12020352

**Published:** 2023-02-08

**Authors:** Maria Baltogianni, Vasileios Giapros, Chrysoula Kosmeri

**Affiliations:** 1 Neonatal Intensive Care Unit, School of Medicine, University of Ioannina, 45500 Ioannina, Greece; 2 Department of Pediatrics, University Hospital of Ioannina, 45500 Ioannina, Greece

**Keywords:** antibiotic resistance, biofilm infections, NICU

## Abstract

Neonatal sepsis is an important cause of neonatal morbidity and mortality. A significant proportion of bacteria causing neonatal sepsis is resistant to multiple antibiotics, not only to the usual empirical first-line regimens, but also to second- and third-line antibiotics in many neonatal intensive care units (NICUs). NICUs have unique antimicrobial stewardship goals. Apart from antimicrobial resistance, NICUs have to deal with another problem, namely biofilm infections, since neonates often have central and peripheral lines, tracheal tubes and other foreign bodies for a prolonged duration. The aim of this review is to describe traditional and novel ways to fight antibiotic-resistant bacteria and biofilm infections in NICUs. The topics discussed will include prevention and control of the spread of infection in NICUs, as well as the wise use of antimicrobial therapy and ways to fight biofilm infections.

## 1. Introduction

Neonatal sepsis is an important cause of neonatal morbidity and mortality. The incidence is 1 to 5 cases per 1000 live births [1,2], and it is the third most common etiology of neonatal mortality [3]. A serious, emerging, worldwide problem is antibiotic resistance, which has been declared a global threat to public health by the Centers for Disease Control and Prevention [4]. Microorganisms normally develop resistance against antibiotics, especially after widespread use of these drugs, by expressing resistant genes that were normally suppressed. Otherwise, microorganisms can acquire resistance genes from other bacteria [5].

A significant proportion of bacteria causing neonatal sepsis is multidrug-resistant (MDR), not only to the usual empirical regimens, but also to second- and third-line antibiotics in many neonatal intensive care units (NICUs) [6,7,8]. A point prevalence study of 226 hospitals in 41 countries from all over the world showed that 40% of the isolated pathogens in neonatal sepsis were resistant to first-line antibiotics that were usually a combination of ampicillin/amoxicillin/benzylpenicillin and aminoglycoside [7]. Another study from four hospitals in Southwest China found that early-onset sepsis (EOS) was most caused by *Escherichia coli*, while the main isolates of late-onset sepsis (LOS) were *Klebsiella pneumoniae* and *Escherichia coli.* Almost 65% of *Escherichia coli* isolates and 78% of LOS isolates were MDR [8]. A cohort study of three tertiary care centers in India found methicillin resistance in 61% of coagulase-negative staphylococci and 38% of *Staphylococcus aureus* isolates [9]. Another study of 183 neonates in India found that almost all isolates of *Pseudomonas aeruginosa* were multidrug-resistant [10].

In NICUs, antibiotics represent the most prescribed drugs and this renders them a high-risk environment for MDR-organism colonization and spread. Furthermore, the prolonged hospitalization of patients and the common use of foreign bodies, such as central and peripheral lines, tracheal tubes and other foreign bodies, are risk factors for antimicrobial resistance and biofilm infections [11].

There is an ongoing effort from several health organizations, such as the Centers for Disease Control and Prevention and the World Health Organization, to address the problem of antibiotic resistance. Many antibiotic stewardship programs are aiming to deal with this problem. However, for common conditions requiring antibiotics in the NICU, there is still a paucity of data to guide day-to-day practice and antibiotic stewardship problems [12].

This review aims to describe traditional and novel ways to fight antibiotic-resistant bacteria and biofilm infections in the NICU. The topics discussed will include prevention and control of the spread of infection in the NICU, the wise use of antimicrobial therapy, and ways to fight biofilm infections.

## 2. Methods

We searched Pubmed and Google Scholar for relevant studies on traditional and novel ways of fighting antibiotic resistance and biofilm infections in NICUs. The following terms when used: “neonatal sepsis”, “neonate”, “infection”, and “antimicrobial resistance”, “multidrug resistance”, “antibiotic resistance”, or “biofilm infections”, up to December 2022. The initial literature search identified almost 1100 relevant studies. The titles, and then abstracts of the retrieved articles, were scanned for relevance. We also reviewed the reference lists of the retrieved articles in search of other relevant articles that could have been missed in the initial search. We included in this narrative review only papers written in English and papers referring to the population hospitalized in NICUs. Finally, 100 articles were included in the main text and specifically systematic reviews, meta-analyses, randomized controlled trials, observational studies, and narrative reviews were included.

## 3. The Unique Characteristics of the Neonatal Population and NICUs

Antimicrobial therapy is a common practice in the everyday routine of NICUs and antibiotics are the most prescribed drugs in NICUs for many reasons. Firstly, neonates, and specifically preterm ones, are more vulnerable to infections and their survival relies on antibiotics. Many risk factors, such as prematurity, low birth weight, and immature immune system, require invasive procedures and prolonged hospitalization, which renders neonates a high-risk group for infection (Table 1).

Moreover, there are many diagnostic challenges in NICUs. Neonatal sepsis can present with non-specific clinical signs, such as apnea, respiratory distress, tachycardia and temperature instability rendering the diagnosis difficult, while on the other hand, the absence of clinical findings does not exclude infection [13]. Many other non-infectious causes can have similar signs and symptoms as sepsis [14]. Consequently, neonates with respiratory distress syndrome, or transient tachypnea of the newborn, are treated with empirical antibiotic therapy until the blood culture results are negative [15,16]. Furthermore, laboratory tests have a low specificity and sensitivity for bacteremia in the early stages. Blood cultures can be falsely negative due to difficulties in obtaining sufficient sample volume, low bacteremia levels and the intrapartum use of antibiotics [17].

The risk factors for developing multidrug-resistant pathogens are also different in NICUs compared to adult and pediatric ICUs. Gestational age below 32 weeks, birth weight below 1500 g and exposure to vancomycin or carbapenems were found to be independent risk factors for methicillin-resistant *Staphylococcus aureus* (MRSA) in NICUs [18,19,20]. Moreover, neonates that were transferred to a tertiary NICU after birth, or were born to mothers colonized with MRSA, also carry an increased risk [18,19,20].

Therefore, NICUs have special antimicrobial stewardship needs that deal with these diagnostic challenges and guide clinical practice.

## 4. Prevention and Control of Infection

A traditional, simple and important way to reduce the spread of resistant bacteria is to reduce the number of infections, since fewer infections means fewer prescribed drugs. Simple and effective methods for infection control, such as safe delivery, avoidance of unnecessary invasive procedures, sanitation, proper cleaning of NICU equipment, hand hygiene before and after interaction with patients or their environment and restricted entry to NICUs are the milestones of prevention and should be strictly adhered to. Hand hygiene is crucial for the restriction of horizontal transfer of MDR microorganisms [21]. Although hand hygiene has long been proven as a very effective measure, a systematic review showed that compliance among healthcare providers is lower than internationally set targets [22]. Vertical transfer of MDR organisms refers to the transmission of such pathogens during labor, rendering safe delivery practices very important [21]. Prompt identification and isolation of MDR-colonized neonates is crucial in the setting of NICUs [11].

Central-line-related infections are also an important problem in NICUs. A prospective registry of 6215 very low birth weight infants found that central line catheters were commonly used in hospitalized infants and were associated with an increased risk of late-onset sepsis [23]. However, central-line-associated bloodstream infections were found to be preventable after proper intervention, as shown both in adult and neonatal ICUs [24,25]. Since line insertion is often mandatory in premature and ill neonates, several prevention strategies have been studied and proved effective in preventing associated infections. Such secondary prevention strategies include proper hand hygiene, appropriate maintenance and handling of central lines, clinician education and prompt removal of unnecessary central lines. The care bundle approach was found to be the most effective one in decreasing central-line-associated infections in various age groups [25,26].

Antifungal prophylaxis is also administered in many NICUs, especially if there is a high incidence of invasive candidiasis. According to the Clinical Practice Guidelines for the Management of Candidiasis in 2016, neonates with a birth weight below 1000 g could be administered intravenous or oral fluconazole twice weekly for 6 weeks. Alternatively, for neonates with a birth weight below 1500 g, oral nystamycin can be used [27].

## 5. Diagnosis of Neonatal Sepsis

NICUs face many diagnostic challenges, as already described. Accurate diagnosis of sepsis and prompt identification of high-risk infants that need antibiotic therapy is an important first step in limiting the inappropriate use of antibiotics. The main challenge is that neonatal sepsis can present with various nonspecific signs and symptoms, while many of these findings overlap widely with those of other noninfectious causes [14]. As a result, cultures are often obtained, and empirical antimicrobial therapy is initiated with a low threshold.

The gold standard for diagnosing sepsis is blood cultures. Ideally, two blood cultures of sufficient blood volume (1–2 mL) should be collected with a sterile technique, before antimicrobial therapy initiation [28,29].

Biomarkers, such as C-reactive protein (CRP), procalcitonin (PCT), white blood cell count and erythrocyte sedimentation rate, can serve as useful adjunctive tools in combination with clinical symptoms and signs in the diagnosis of infection. A systematic review and meta-analysis of 22 cohort studies including 2255 infants, found that CRP was not helpful at the initial evaluation of possible LOS, and was insufficient alone to guide diagnostic and treatment decisions [30]. However, the combination of PCT and CRP or presepsin alone improved the accuracy of diagnosis of neonatal sepsis, as shown in a meta-analysis of 28 studies enrolling 2661 neonatal patients [31]. Randomized controlled trials have tested the accuracy and efficacy of PCT. The use of serial PCT values, in the decision tree process, led to a shorter duration of antibiotic therapy in EOS [32,33]. Interleukin-6 (IL-6) is another potentially useful marker that has been studied in neonatal sepsis. A systematic review of 3276 newborns found that IL-6 is a good early diagnostic marker of EOS, with higher accuracy when used in preterm neonates and when measured in the first 48 h [34]. The sensitivity had a wide range of 42.1–100%, and a specificity of 43–100% (with median values of 83 and 83.3%, respectively). A meta-analysis of 694 neonates showed higher sensitivity and specificity of IL-6 (pooled sensitivity 85% and specificity 88%) in infants with premature rupture of membranes [35].

Another area of active research is the development and validation of sepsis calculators to better predict EOS. In 2014, Escobar G et al. published a retrospective nested case-control study of 600,000 neonates from 14 hospitals from 1993 to 2007, and developed a risk classification scheme for EOS. This calculator, which is perhaps the most promising and widely used, applies to neonates 34-week-old and older and is a method of estimating the risk of EOS. This calculator uses both maternal risk factors, as well as factors from the clinical examination of the newborn, and the neonates are stratified into three risk groups: treat empirically, observe and evaluate, and continued observation [36,37].

The accuracy of this calculator, provided by the Kaiser Permanente Division of Research, has been tested in many studies. A meta-analysis of 13 studies, analyzing a total of 175,752 newborns, showed that the use of this calculator limited the use of antibiotic therapy for suspected EOS without missing cases of EOS, while evidence regarding safety was limited [38]. The number of missed cases of EOS was similar, whether the EOS calculator was used or conventional management strategies were applied [38]. A retrospective study from our center found that the adoption of the online sepsis risk calculator would have significantly reduced antibiotic usage, however, a significant proportion of the cases would have been missed [39]. Therefore, the calculator can be applied to late preterm and term neonates with EOS, but it is not useful for preterm neonates.

For LOS, there is a score, namely, the RALIS score [40,41], that is based on vital signs, including heart rate, respiratory rate, body temperature, oxygen saturation below 85%, bradycardia and body weight, monitored 12 times a day. The RALIS score has the advantage of being a non-invasive tool and uses clinical data that are routinely collected in NICUs [41]. This score has been tested in a prospective study of 118 very low birth weight infants and was found to have reasonable sensitivity (75%) and specificity (81%), and its negative predictive value was very high (95%). However, it had a low positive predictive value of almost 39% [41]. A smaller retrospective study of 73 preterm infants found higher sensitivity (82%) for the RALIS score, but low specificity (44%) and a better positive predictive value (67%) [40]. In both studies, the RALIS alert preceded clinical suspicion by almost 3 days. The RALIS score is a promising tool when used in association with clinical judgement, laboratory values and specific infection biomarkers.

Except for the RALIS score, there is a variety of predictive scores for LOS that has been studied in the literature. The available scores were based exclusively on clinical variables, laboratory variables or risk factors, or were based on combinations of these variables. Sofouli et al. conducted a systematic review of the available scores and concluded that the majority of them may assist in early diagnosis, but almost all had a limited diagnostic accuracy [42]. The scores that were found to have high sensitivity and negative predictive value were those based on clinical, laboratory and risk factors, such as the NOSEP-NEW-I and NOSEP-NEW-II scores [42,43], and on clinical, laboratory variables and management decisions [42,44,45].

Therefore, accurate diagnosis of sepsis is of paramount importance in order to restrict antibiotic administration. A combination of clinical signs and symptoms, properly drawn blood cultures and serum biomarkers can help promptly identify suspected sepsis. The sepsis calculators are also adjunct useful tools. Serum biomarkers are also useful in limiting the duration of antibiotic therapy.

## 6. Prudent Antibiotic Treatment

Once the clinical decision that antibiotic therapy should be initiated has been made, the wise selection of antibiotics is mandatory. Antibiotics are the most prescribed drugs in the NICU and account for one-third of the most frequently used medications [46,47]. The most common causes of EOS are group B *Streptococcus* and *Escherichia coli*, while LOS is also caused by other Gram-negative bacilli, *Candida* spp., and *Staphylococcus* [2,23]. Empirical antibiotic therapy should be effective against these common pathogens. In EOS, the regimen of ampicillin and gentamicin is usually used despite an increase in ampicillin resistance [48].

In LOS, there is a wide variation of antibiotic use among centers and NICUs. In a study of ten units in the Netherlands, seven different empiric regimens were used for LOS, six for meningitis, and seven for necrotizing enterocolitis (NEC) [49]. A retrospective study of eleven Greek NICUs, including 418 infants, found that the most common regimen for EOS was ampicillin with gentamicin. On the contrary, for LOS, there was a wide variation of different antimicrobial combinations used, with the most common variation being meropenem with a glycopeptide and piperacillin/tazobactam with a glycopeptide [50]. The wide variation in antibiotic therapy used for LOS has also been documented in a multicenter study of six NICUs in Australia [51], as well as in a prospective, observational study from five European countries [52].

The most widely recommended regimen for LOS is the combination of a semisynthetic penicillin with an aminoglycoside. For Gram-negative organisms, piperacillin-tazobactam or cefepime can be used instead, especially in infants known to be colonized with resistant Gram-negative organisms [53]. Third-generation cephalosporins should be used only in cases when there is a suspicion of meningitis, because of the increased risk of resistance and invasive candidiasis, particularly in the low birth weight group [53,54,55]. Vancomycin is commonly administered as empiric therapy in many NICUs, since coagulase-negative staphylococci are found to be resistant to antistaphylococcal penicillins. This is a problem and many NICUs permit the use of this antibiotic only if there is an isolation of resistant, coagulase-negative staphylococci in blood cultures, and avoid using this glycopeptide as empiric treatment. The implementation of a “NICU vancomycin-use guideline” reduced the exposure of newborns to vancomycin with no adverse effect on neonatal morbidity and mortality [56]. Moreover, when there is a low incidence of methicillin-resistant *Staphylococcus Aureus*, vancomycin usage should also be reserved and nafcillin or oxacillin should be preferred instead. The most commonly used antibiotic regimens in neonatal sepsis are shown in Table 2.

For NEC there are no guidelines to clearly state the optimal empirical therapy. The empirical regimen should include antibiotics that are effective against the most common pathogens associated with NEC, but also preserve the normal microbiota [29]. Studies showed a wide variety of antibiotic agents for the treatment of NEC. In 90 patients that had surgical NEC, the most common regimen was ampicillin, gentamicin and metronidazole, while 22 different antibiotic combinations were used pre-operatively [59]. It is worth mentioning that no regimen was found to be superior. Another study of 160 patients with NEC also demonstrated the variability of regimens used since 14 different antibiotic combinations were identified [58]. This study also showed that patients that had medical NEC received mostly ampicillin, gentamicin, and metronidazole, while patients with surgical NEC received vancomycin and antipseudomonal agents [60].

A wide variability in antimicrobial therapy was also identified in neonates that needed surgical intervention. In a study of 191 neonates with gastroschisis, some sites used the combination of ampicillin and gentamicin, while other sites used the combination of cefotaxime and metronidazole [61].

The high variability of antibiotic regimens in common neonatal condition, such as LOS and NEC, highlights the need for treatment guidelines for these conditions. The goal of antimicrobial stewardship programs is to optimize antimicrobial prescribing habits. To limit the risk of emerging antibiotic-resistant microbes, the narrowest spectrum antibiotic regimen should be given for the shortest duration of time. Longer antibiotic courses are associated with an increased risk of colonization with multidrug-resistant organisms, necrotizing enterocolitis, and/or death [62,63]. Continuous epidemiological surveillance of responsible pathogens and their antimicrobial resistance patterns is very important, at both local and national levels, for the best narrow-spectrum antibiotic selection.

Furthermore, in newborns, it is difficult to administer the optimal dose of antimicrobials and accurately monitor the drug levels since the glomerular filtration rate is lower and drugs exhibit different pharmacodynamics and pharmacokinetics [12].

## 7. De-Escalation of Therapy

It is very important that antibiotic administration is constantly reevaluated and ceased when there is no further indication. When the culture results become available within 48 h, the antimicrobial therapy should be modified according to the isolated pathogen and its sensitivities. This practice is described as a de-escalation of therapy [64]. Specifically, antibiotics should be changed to the narrowest-spectrum agent that has activity against the pathogen [53,65]. Although this strategy seems logical and has many advantages, it is often not followed. Therefore, the failure of de-escalation of antibiotic therapy in NICUs is considered one of the most common causes of inappropriate antimicrobial use [12,53]. Moreover, antibiotics should be discontinued if the cultures remain sterile at 48 h, provided that the infant is improving clinically [48].

Limiting patient exposure to broad-spectrum antibiotics can result in decreased antimicrobial resistance and cost [66]. Furthermore, in premature infants with sterile cultures in the first week of life, prolonged administration of empiric antibiotics was associated with an increased length of hospitalization, incidence of NEC, and death [67].

While step-down or de-escalation of therapy is common advice in stewardship programs, it is often not followed, since clinicians tend to continue the treatment that was proven to be effective [18,68].

## 8. Treatment Duration and Prompt Discontinuation of Therapy

The duration of empiric treatment for possible EOS in the NICU varies significantly [17,69,70]. A prospective study of 15 public NICUs in Greece showed that the median duration of antibiotics ranged from 2 to 8 days [71]. Another study of three NICUs in the USA showed that the median duration of antibiotics was 7 days, and there was a range of 5 to 14 days for cases of pneumonia, despite sterile cultures and cases of culture-negative sepsis [72].

The duration of antibiotic therapy should be well established for many clinical conditions, such as sepsis, NEC, and perioperative prophylaxis, and should be constantly re-evaluated for proper discontinuation [29]. For NEC, an antibiotic course of 7 to 14 days is often recommended. Additionally, some studies have found similar patient outcomes with shorter regimens of 7 to 10 days compared to longer regimens, further supporting the need for limiting antibiotic duration [18,58,73].

In asymptomatic newborns born to mothers with chorioamnionitis, the American Committee of Fetus and Newborn recommends a discontinuation of antibiotics within 48 to 72 h, if blood cultures remain negative [12,74]. If there are abnormal laboratory results and negative blood cultures, the antibiotics could be continued for a further 72 h [74,75].

For other neonatal infections, the recommended antimicrobial duration is 7 to 10 days for hospital-acquired pneumonia, 10 to 14 days for uncomplicated bloodstream infection, 14 to 21 days for complicated bloodstream infections, 7 to 14 days for catheter-associated UTI and 48 to 72 h for culture-negative chorioamnionitis [18]. Regarding neonatal pneumonia, a 4-day antibiotic course, followed by a 24-h observation phase, was found comparable to a 7-day course when neonates were asymptomatic after 48 h of treatment initiation [76]. Generally, there are no consensus guidelines and there is controversy regarding the appropriate duration of treatment [18].

The duration of therapy for culture-negative sepsis varied widely in a retrospective study of 30 academic NICUs, while the duration of the antibiotic course was not associated with clinical signs or antepartum risk factors for sepsis [77]. An examination of the PROACT database of Paradigm Health of term infants found that even though all infants were clinically well and blood cultures were sterile, 17% of infants were treated for 4 to 6 days and almost 12% were treated for 7 to 10 days [78]. A study from 15 Greek NICUs, including our tertiary NICU, found that the duration of antimicrobial therapy can be reduced in low-risk infants for EOS, following the “low-hanging fruit approach”. This antibiotic stewardship intervention included discontinuation of the antibiotics by day 5 for neonates of gestational age ≥ 37 weeks, absence of signs or symptoms of sepsis, CRP ≤ 10 mg/L and negative cultures within 3 days of antibiotic initiation [71].

There is a scarcity of data regarding the duration of perioperative chemoprophylaxis in neonates undergoing surgery. A European study of 1799 children showed that surgical prophylaxis was administered for more than 1 day in 67% of children and neonates [79]. In a study of 191 neonates with gastroschisis, the duration of antibiotic prophylaxis varied widely between sites, ranging from less than 3 to more than 14 days [61]. Another retrospective, single-center study of 74 infants with abdominal wall defects showed that all infants received antibiotic prophylaxis of at least 1 day duration [80]. There is no clear evidence that there is any benefit of perioperative prophylaxis continuation for more than 24 h postoperatively [29]. It is also worth mentioning that there are operations where prophylactic antibiotics are not required, such as neonatal testicular torsion, inguinal hernia, gastroschisis, omphalocele and laparoscopic pyolorotomy [81].

Antibiotic stewardship efforts should focus on standardizing the duration and the empirical therapy of common neonatal conditions, to prevent antibiotic resistance, since longer antimicrobial therapy duration favors the development of resistant pathogens. The summary of the available ways to fight antibiotic resistance is shown in Table 3.

## 9. Fighting Biofilm Infections

Biofilm formation is a severe health concern in NICUs and a persistent threat, as it increases both morbidity and mortality. Bacterial biofilms are immobile microbial communities with diverse bacterial cell colonies in groups, which colonize and grow on the surfaces of almost all medical devices or prostheses [82]. The development of biofilm has many stages, including attachment of macro and micro molecules to surfaces, colonization and proliferation of bacterial pathogens, the release of extracellular polymeric substances and biofilm maturation and detachment [83,84,85].

It has been estimated that most bacterial infections in humans are correlated with biofilms and about 50% of the nosocomial infections are indwelling-device associated [86]. Neonates hospitalized in NICUs are at a high risk of biofilm infections, since they often need central lines, endotracheal tubes, umbilical arterial and vein catheters and peripheral central lines for a prolonged duration. Bacterial biofilms are highly resistant to antibiotic treatments and immune responses [87]. Biofilms provide protection to bacteria from the pH, nutrient deficiency and mechanical forces, and also prevent antibiotic and host immune cells from gaining access to their bacteria [83,84,88]. Therefore, biofilms contribute to persistent and resistant chronic infections.

Bacteria that have been associated with biofilm infections in central venous catheters are *Enterococcus faecalis*, *Pseudomonas aeruginosa*, *Staphylococcus aureus* and *Staphylococcus epidermidis*, while numerous other bacteria have been associated with biofilm infections in other adherent surfaces [83].

Although it is well known that antibiotic treatment is currently the most important and effective measure of control for microbial infections, antibiotic treatments are almost impossible to eradicate biofilm infections [86]. The development of intact biofilms is critical for the spreading and persistence of bacterial infections in the host [84]. Therefore, biofilms give the power to bacteria to become resistant to antibiotics.

As already described, biofilm infections are chronic and can have exacerbations. Antimicrobial therapy can be helpful in mitigating an exacerbation, but cannot fully eradicate the bacteria in a biofilm [86]. Since biofilms are multimicrobial, antibiotic therapy should cover against Gram-positive, Gram-negative and fungal microorganisms, as well as resistant bacteria. The above facts render treatment of a biofilm infection really challenging. Antibiotics are more effective when used in the early stages of biofilm formation, probably because the cells are not completely embedded into biofilm communities [83,89,90]. The antibiotic regimens should have a wide spectrum and employ different modes of action. Removal of the foreign body or replacement of the central venous catheter is almost always mandatory to successfully control a biofilm infection [86]. A short course of intravenous antibiotic treatment is also important to eradicate the bacteria that were released into the bloodstream.

There are many novel approaches under development for the prevention and treatment of biofilm infections. Specific agents may inhibit the initial biofilm attachment, result in the removal of biofilms or inhibit biofilm formation by quorum quenching (the communication process among cells) [91]. Initial biofilm attachment can be inhibited by altering either the chemical or physical properties of the biomaterials. For example, antibiotics, biocides and ion coatings modify the surface of devices and inhibit biofilm formation [92]. Moreover, hydrophilic polymers, such as hyaluronic acid and poly N-vinylpyrrolidone, alter the hydrophobicity of polymeric materials and reduce the adhesion of microbes [93,94]. This occurs in the same way by altering the physical properties of devices, various superhydrophobic surfaces, hydrogel coatings and heparin coatings [95,96]. Another studied field is the novel ways to remove biofilms. Matrix-degrading enzymes have the purpose of dissociating a biofilm matrix and allowing antimicrobials to act more drastically [97]. Furthermore, free fatty acids, specific amino acids and nitric oxide generators have been shown to induce biofilm dispersal against several bacteria [98,99]. Quorum sensing is crucial in biofilm formation, therefore several strategies have been studied to inhibit this system of cellular communication in biofilm [91]. Molecules that degrade, inhibit or antagonize quorum sensing signals have been studied, as well as molecules that inhibit signal transduction or transportation [91]. These novel approaches could lead to anti-biofilm therapies in the future, that are much more effective than current antibiotic treatments [83,84,86,100].

At present, the prudent use in the NICU of all those foreign materials that act as substrates for biofilm infections is very important. For example, catheters should be removed as soon as possible, and less invasive ventilation modalities should be used (i.e., nCPAP instead of intubation). Traditional and novel ways of fighting antibiotic resistance are shown in Table 4.

## 10. Conclusions

The neonatal population has unique characteristics and antibiotic stewardship needs in order to restrict antibiotic resistance and biofilm infections. Simple and effective methods for infection control should be strictly followed by clinicians, and are milestones in the prevention of infections. Accurate diagnosis of sepsis, use of the narrowest-spectrum of antibiotics and for the shortest necessary duration, as well as de-escalation and proper discontinuation of therapy are important steps in the wiser use of antibiotics, that will limit antibiotic resistance. Lastly, biofilm infections are very difficult to treat and therefore, proper handling and early withdrawal of foreign materials is mandatory in order to prevent them.

## Figures and Tables

**Table 1:** Main risk factors for neonatal infection.

Prematurity
Low birth weight
Immature immune system
Admission to NICU and need for invasive procedures
Prolonged hospitalization
Transferred to a tertiary NICU after birth
Previous exposure to antibiotics (specifically vancomycin or carbapenems)
Born to mothers colonized with MRSA
Foreign bodies (central-line catheters, indwelling catheters), mechanical ventilation

MRSA: methicillin-resistant *Staphylococcus aureus*, NICU: neonatal intensive care unit.

**Table 2:** Most common antibiotic regimens used in neonatal sepsis [23,48,53,56,57,58].

Condition	Most Common Pathogen	Antibiotic Regimen
EOS (age < 72 h)	Empiric therapy	Ampicillin plus aminoglycoside
LOS (age ≥ 72 h)	Empiric therapy	Ampicillin plus aminoglycoside or Ampicillin plus cefepime
Culture-proven sepsis	Group B streptococcus	Penicillin
	*Escherichia coli*	Ampicillin or expanded-spectrum cephalosporin in resistant strains
	Gram (-) bacilli	Ampicillin and gentamicin or cefepime or piperacillin/tazobactam
	*Listeria monocytogenes*	Ampicillin plus gentamicin
	Coagulase negative Staphylococci	Antistaphylococcal penicillin * Vancomycin in resistant strains
	*Staphylococcus aureus*	Nafcillin or oxacillin* Vancomycin in resistant strains
	*Candida* spp. (invasive infection)	Amphotericin B or fluconazole

EOS: early-onset sepsis, LOS: late-onset sepsis.

**Table 3:** Ways of fighting antibiotic resistance in NICU.

Prevention of infection Safe delivery Hand hygiene Avoidance of unnecessary invasive procedures Sanitation Proper cleaning of equipment Restricted entry to NICU Proper maintenance and handling of central lines
Prompt diagnosis of sepsis Proper draw of blood cultures (at least two blood cultures before treatment initiation, adequate volume, sterile technique) Use of biomarkers (CRP, PCT, Il-6) Use of sepsis calculators for EOS and LOS
Wise antibiotic treatment Empiric therapy that is effective against the most common pathogens Use of third-generation cephalosporins only in suspicion of meningitis Use of vancomycin only in proven resistant staphylococci
De-escalation of therapy with availability of culture results Use the narrowest-spectrum antibiotic regimen
Appropriate treatment duration
Prompt discontinuation of therapy

CRP: c-reactive protein, EOS: early-onset sepsis, Il-6: interleukin-6, LOS: late-onset sepsis, NICU: neonatal intensive care unit, PCT: procalcitonin.

**Table 4:** Traditional and novel ways of fighting biofilm infections.

Traditional ways Early removal of foreign bodies when possible Replacement of central venous catheters when mandatory Use of antibiotics Use of less invasive ventilation modalities
Novel ways Inhibition of initial biofilm attachment Removal of biofilms Quorum sensing system blockage

## Data Availability

Not applicable.
